# Impacts of vitamin premix and/or yeast ingredient inclusion in a canned cat food on thiamin retention during 6 months of storage

**DOI:** 10.3389/fvets.2022.1090695

**Published:** 2022-12-08

**Authors:** Amanda N. Dainton, Brittany White, Leah Lambrakis, Charles Gregory Aldrich

**Affiliations:** ^1^Department of Grain Science and Industry, Kansas State University, Manhattan, KS, United States; ^2^Simmons Pet Food, Inc., Siloam Springs, AR, United States

**Keywords:** canned cat food, degradation, retention, storage, thiamin, vitamin B1, wet cat food, yeast

## Abstract

**Introduction:**

Low thiamin levels in thermally processed canned cat foods are concerning for the pet food industry. However, there is little information on storage stability of thiamin in this food format or if inclusion of select ingredients, such as dried yeasts, has an effect. Therefore, the objective was to evaluate the storage stability of thiamin when a vitamin premix and/or yeasts ingredients were included in a canned cat food.

**Materials and methods:**

The factorial treatment arrangement consisted of 2 levels of vitamin premix (with or without) and 4 inclusions of yeast (NY = none, LBV = Lalmin B Complex Vitamins, BY = product #1064B, or EA = BGYADVANTAGE). Diets were stored for 6 months and analyzed every month for thiamin. Data were analyzed as a mixed model (SAS v. 9.4; SAS Institute, Cary, NC) with fixed effects (vitamin premix, yeast, time, and their two-way and three-way interactions) and random effects (production day and the interaction of production day, vitamin premix, and yeast). Significance was set at *P* < 0.05 and Fisher's LSD was used to separate means.

**Results and discussion:**

Diets including the vitamin premix [average 55.1 mg/kg dry matter basis (DMB)] contained more (*P* < 0.05) thiamin than diets that did not (average 7.5 mg/kg DMB). Inclusion of LBV (average 40.3 mg/kg DMB) resulted in the highest (*P* < 0.05) levels of thiamin, followed by BY (*P* < 0.05; average 26.9 mg/kg DMB). Diets with NY and EA contained the lowest (*P* < 0.05) levels of thiamin and were not different from each other (*P* > 0.05; average 19.3 mg/kg DMB). The diet containing vitamin premix without yeast lost (*P* < 0.05) 17.8% thiamin while diets containing a yeast ingredient maintained thiamin levels better during storage. This suggested that thiamin from yeast ingredients was more resistant to degradation during storage and should be considered when designing new canned cat foods.

## Introduction

Thiamin is a water-soluble B-vitamin. It is found in plants in its free form (molecular formula: C_12_H_17_N_4_OS^+^) and in animals as a phosphorylated form, typically thiamin diphosphate [also called thiamin pyrophosphate; molecular formula: C_12_H_19_N4O_7_P_2_S+; (1)]. Thiamin can be produced synthetically as thiamin mononitrate (molecular formula: C_12_H_17_N_4_OS·NO_3_) or thiamin hydrochloride (molecular formula: C_12_H_17_N_4_OS^+^Cl^−^·HCl). Once thiamin is consumed, it is digested to the free form, absorbed in the small intestine, and the majority is phosphorylated in the liver (1). Thiamin is a critical coenzyme for the conversion glucose to pyruvate, pyruvate to acetyl-CoA, and α-ketoglutarate to succinyl-CoA as well as for the oxidation of branched α-keto acids. Additionally, thiamin is involved in normal functions of the nervous system (1).

Due to the water solubility of thiamin, excesses of the vitamin cannot be stored in the body and are excreted in urine (1). This poses a risk for cats, who have a high thiamin requirement of 5.6 mg of thiamin per kg of diet on a dry matter basis [DMB; (2)]. Cats who do not consume diets with sufficient levels of thiamin may exhibit instability, decreased appetite, ventroflexion, and seizures among other clinical symptoms (3–6). Prolonged deficiency can lead to permanent brain damage that affects learning (7) and death within a month. As such, achieving sufficient levels of thiamin in foods for cats after processing and throughout the product's shelf life is critical.

Many cats in the United States are fed canned foods that undergo thermal processing with a retort. This is a regulated process to commercially sterilize food under high pressure and high temperatures (8). Canned foods contain high levels of moisture and experience prolonged heat exposure, both of which often result in decreased thiamin levels post-processing. Many researchers have addressed factors that can influence thiamin degradation due to thermal processing (9–11). A previous research paper evaluated two dried brewer's yeasts (spray dried brewer's yeast #1064B, abbr. BY; BGYADVANTAGE, abbr. EA) and a fortified inactive yeast (Lalmin B-Complex Vitamins, abbr. LBV) for their thiamin stability in a canned cat food through thermal processing. This research identified a spray-dried brewer's yeast (BY) with similar thermal degradation levels compared to thiamin mononitrate, the standard thiamin source for canned cat foods, and a fortified inactive yeast ingredient (LBV) with high levels of thiamin (12). While these were meaningful findings, previous research in extruded pet foods found that thiamin levels decreased during storage of the diet (13). As it stands, there are no published values for thiamin degradation during storage in a canned cat food, let alone in canned cat foods containing either of the previously mentioned yeast ingredients.

The objective of this experiment was to determine the effect of 6 months of storage on thiamin content of canned cat foods containing a yeast ingredient and/or a thiamin mononitrate from a vitamin premix. The hypothesis was that inclusion of a yeast ingredient would improve the storage stability of dietary thiamin compared to thiamin mononitrate.

## Materials and methods

### Experimental treatment production

The experimental treatments and their production have been described previously (12). Briefly, 8 experimental treatments were arranged as a factorial with 2 categorical levels of vitamin premix (no vitamin premix or 0.08% vitamin premix containing thiamin mononitrate) and 4 categorical levels of dried yeast [no yeast (NY), 0.65% LBV, 5.00% BY, or 5.00% EA; Table 1]. The 0.08% vitamin premix inclusion was chosen to simulate a commercial canned cat food with around 23 times the AAFCO minimum level of thiamin for adult cats supplied by the vitamin premix prior to diet processing. The level of LBV (Lallemand Bio-Ingredients, a division of Lallemand Inc., Montréal, QC, CA) was chosen to match the thiamin supplied by the vitamin premix. However, formulation levels were based on a yeast ingredient screening analysis and the LBV used in experimental treatment production contained less thiamin than expected (12). The levels for BY (The Peterson Company, Kalamazoo, MI) and EA (The F.L. Emmert Company, Cincinnati, OH) were capped at 5.00% for practicality as levels >5.00% could impose processing challenges.

**Table 1:** Ingredient composition of canned cat foods containing different levels of a vitamin premix and/or a dried yeast ingredient^a^.

	**No vitamin premix**				**Contains vitamin premix**			
**Ingredient, % as-is**	**NY**	**LBV**	**BY**	**EA**	**NY**	**LBV**	**BY**	**EA**
Basal batter^b^	94.92	94.92	94.92	94.92	94.92	94.92	94.92	94.92
Ground brewer's rice	5.08	4.43	0.08	0.08	5.00	4.35	-	-
Vitamin premix	-	-	-	-	0.08	0.08	0.08	0.08
Yeast ingredient	-	0.65	5.00	5.00	-	0.65	5.00	5.00

a NY, no yeast; LBV, Lalmin B-Complex Vitamins; BY, spray-dried brewer's yeast #1064B; EA, BGYADVANTAGE.

b 1 kg of basal batter contains 283.48 g mechanically deboned low ash chicken, 230.33 g frozen pork liver, 223.59 g water, 148.83 g ground chicken, 106.30 g steam, 3.19 g guar gum, 1.35 g potassium chloride, 1.06 g mineral premix for cats, 0.74 g kappa carrageenan, 0.57 g taurine, 0.53 g salt, and 0.02 g 50% vitamin E.

All 8 diets were produced each day for 3 consecutive days in a commercial pet food cannery. All ingredients except the brewer's rice, vitamin premix, and yeast ingredients were prepared, mixed into a basal batter, and heated daily to 50°C daily. Then, the respective brewer's rice, vitamin premix, and/or yeast ingredient were added to 7.6 kg of the basal batter and blended for 1 min while maintaining temperature at 50°C to create each experimental treatment. Aliquots of 156 g of the final batter were transferred to cans (size 307 × 109; Crown Holdings, Philadelphia, PA), which were subsequently closed (Pneumatic Scale Angelus, Stow, OH) with an easy-open lid. All experimental treatments produced on the same day were processed in a horizontal steam batch retort (Versatort Multimode 1520; Allpax, Covington, LA) at the same time. The process schedule, or the thermal conditions utilized to effectively eliminate microorganisms that reproduce in food stored under ambient conditions and microorganisms and spores that pose a public health risk (8), consisted of a 10 min come-up cycle, a 63 min cooking cycle with a target temperature of 123°C, and a 27 min cooling cycle. After the process schedule was complete, cans were removed from the retort and cooled to room temperature. This process created 8 canned diets to serve as experimental treatments for the storage study with ranging thiamin contents. Ranking the experimental treatments by their average thiamin contents immediately after processing appears as follows: NY without the vitamin premix (0.7 mg/kg DMB), EA without the vitamin premix (2.2 mg/kg DMB), BY without the vitamin premix (5.8 mg/kg DMB), LBV without the vitamin premix (19.9 mg/kg DMB), EA with the vitamin premix (46.5 mg/kg DMB), NY with the vitamin premix (54.7 mg/kg DMB), LBV with the vitamin premix (59.6 mg/kg DMB), and BY with the vitamin premix [60.7 mg/kg DMB; (12)].

### Storage of processed diets

Three cans from each combination of experimental treatment and production day were submitted to a commercial lab (Midwest Laboratories; Omaha, NE) for nutritional analysis. This timepoint was considered as “Month 0.” All other cans were stored in a commercial pet food warehouse (Emporia, KS) for 6 months. This length was chosen because canned pet foods may take 2–6 months from diet production to be purchased by a pet owner. Therefore, it is important that thiamin degradation is minimal during this time. Storage took place from October 2020 to April 2021. Temperature was not controlled but never fell below 4.4°C. Diets were stored in their original sealed cans.

### Chemical analyses

A composite of three cans of each treatment replicate was analyzed monthly for the duration of diet storage by a commercial laboratory (Midwest Laboratories; Omaha, NE). Treatment replicate composites were analyzed in duplicate for moisture (AOAC 930.15), thiamin (AOAC 942.23), crude protein (AOAC 99003), crude fat (AOAC 954.02), crude fiber (AOCS Ba 6a-05), ash (AOAC 942.05), and minerals (sulfur, phosphorus, potassium, magnesium, calcium, sodium, iron, manganese, copper, and zinc; AOAC 985.01). Results below the minimum detection level were treated as zeros and duplicate values were averaged for one value per sample. Nitrogen free extract (NFE) was calculated by subtracting the DMB contents of crude protein, crude fat, crude fiber, and ash from 100%.

### Statistical analysis

Macronutrient (moisture, crude protein, crude fat, crude fiber, ash, and NFE) and mineral contents were averaged across all 7 time points for each of the 8 diets produced. Values for these nutrients were presented as mean values ± one standard deviation. This was chosen rather than conducting an analysis of variance (ANOVA) because previous research did not find meaningful differences in moisture, nitrogen, crude fat, and ash contents of a pet food consisting of chunks in gravy, jelly, or water stored for 28 days (14).

Thiamin contents of diets were transformed using the square root function to meet the model assumptions of normality and equal variances. Data were analyzed as a split-plot in time with the fixed effects of vitamin premix, yeast, and time and the random effects of production day and production day by vitamin premix by yeast. Significance of the main effects (vitamin premix, yeast, and time) and all 2-way and 3-way interactions were determined with an ANOVA. Denominator degrees of freedom were adjusted using the Kenward-Roger adjustment. Means were separated using the Fisher's least significant difference (LSD). Single degree of freedom contrasts were analyzed on the main effect of time to determine if the change in thiamin content followed a linear, quadratic, or cubic relationship. Significance for the ANOVA, means separation, and single degree of freedom contrasts was set at α = 0.05. The GLIMMIX procedure was employed in this analysis (SAS v. 9.4; SAS Institute, Cary, NC). Data were presented as back-transformed mean values with 95% confidence intervals (CI).

## Results

### Average macronutrient and mineral contents

Macronutrients were relatively unaffected by ingredient composition and storage time (Supplementary Table 1). Average moisture content ranged from 82.1% (EA with and without vitamin premix) to 83.1% (BY without vitamin premix). Crude fat (average minimum 26.9% DMB for BY without vitamin premix; average maximum 30.9% DMB for NY with vitamin premix), crude fiber (average minimum 0.67% DMB for LBV without vitamin premix; average maximum 1.83% DMB for EA with vitamin premix), and ash (average minimum 5.51% DMB for NY without vitamin premix; average maximum 7.70% DMB for EA with vitamin premix) were more spread out with greater variation. Crude protein exhibited the widest range with an average minimum of 38.2% DMB (NY without vitamin premix) and an average maximum of 51.0% DMB (BY with vitamin premix). Contents of NFE exhibited a similar range width; on average the minimum NFE was observed in EA with vitamin premix (10.5% DMB) and the maximum NFE observed in NY without vitamin premix (25.5% DMB).

Similarly, average mineral contents of the 8 diets during 6 months of storage were close when variation was considered (Supplementary Table 1). Notable ranges were observed for calcium (average minimum 0.96% DMB for BY without vitamin premix; average maximum 1.46% DMB for EA with vitamin premix), magnesium (average minimum 0.072% DMB for NY without vitamin premix; average maximum 0.142% DMB for EA with vitamin premix), iron (average minimum 241 mg/kg DMB for BY without vitamin premix; average maximum 293 mg/kg DMB for EA with vitamin premix), copper (average minimum 32.59 mg/kg DMB for LBV with vitamin premix; average maximum 39.23 mg/kg DMB for BY without vitamin premix), and manganese (12.69 mg/kg DMB for BY without vitamin premix; average maximum 26.38 mg/kg DMB for EA with vitamin premix).

### Thiamin content during storage

**Figure 1:**
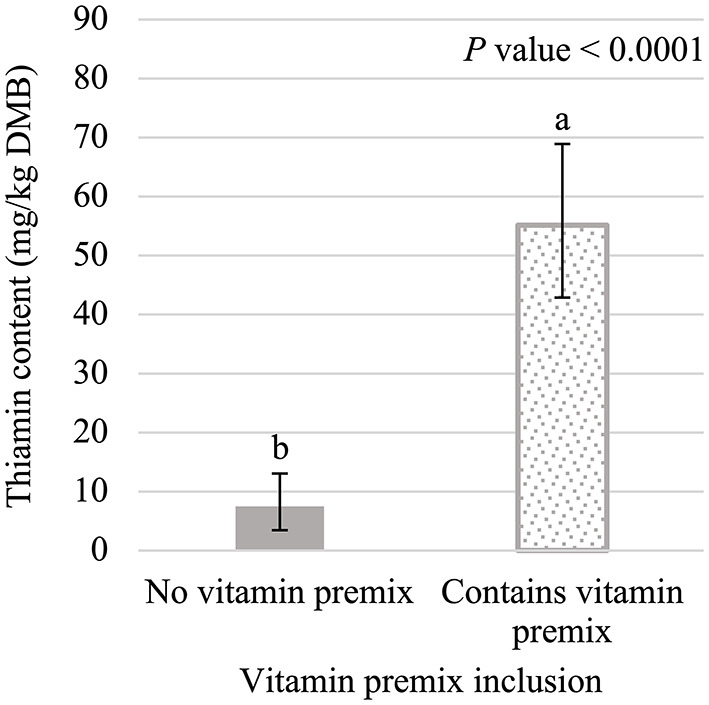
Main effect of vitamin premix inclusion on dry matter basis (DMB) thiamin content (average with 95% confidence interval) of canned cat foods stored in a commercial warehouse for 6 months. ^a,b^Means without a common superscript are different (*P* < 0.05).

**Figure 2:**
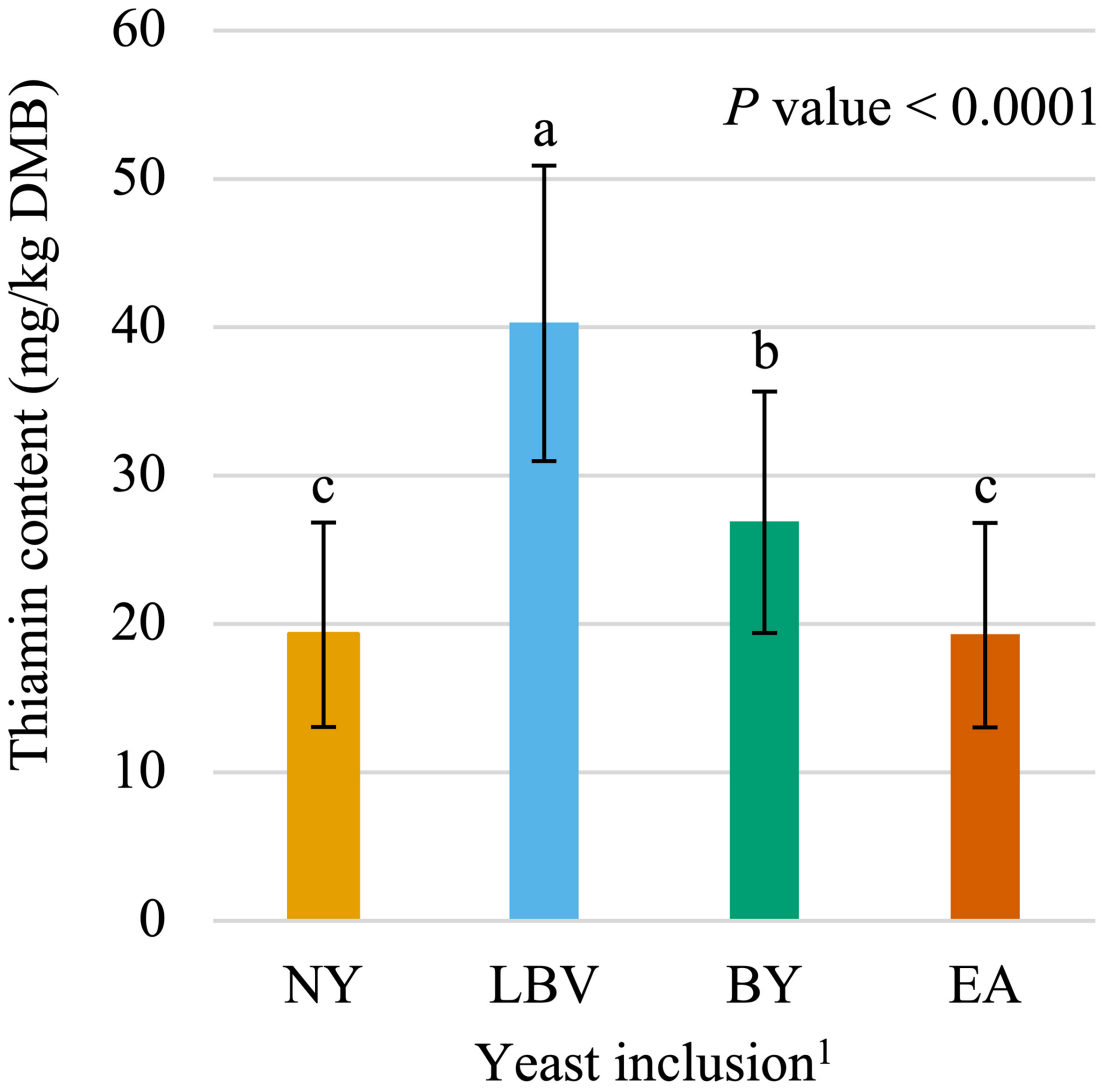
Main effect of yeast inclusion^1^ on dry matter basis (DMB) thiamin content (average with 95% confidence interval) of canned cat food stored in a commercial warehouse for 6 months. ^abc^ Means without a common superscript are different (*P* < 0.05). ^1^NY, no yeast; LBV, Lalmin B-complex vitamins; BY, spray dried brewer's yeast #1064B; EA, BGYADVANTAGE.

**Figure 3:**
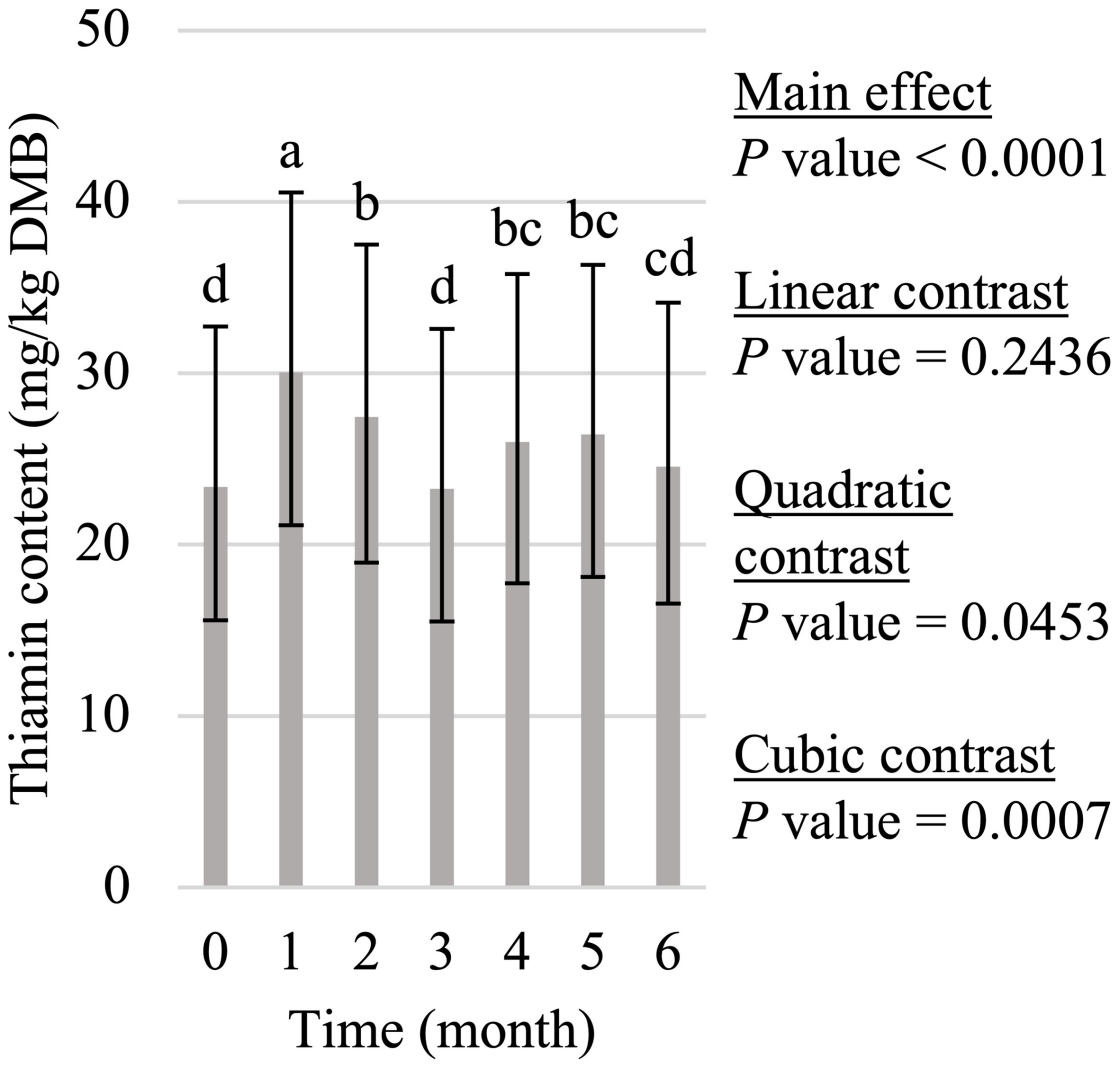
Main effect of time on dry matter basis (DMB) thiamin content (average with 95% confidence interval) of canned cat food stored in a commercial warehouse for 6 months. ^a−d^Means without a common superscript are different (*P* < 0.05).

The main effects of vitamin premix (*P* < 0.05; Figure 1), yeast (*P* < 0.05; Figure 2), and time (*P* < 0.05; Figure 3) were all significant. Thiamin content when the vitamin premix was included in the formula (55.1 mg/kg DMB; 95% CI = 42.9 mg/kg DMB, 68.9 mg/kg DMB) was 6.4 times higher (*P* < 0.05) than when the vitamin premix was left out of the formula (7.5 mg/kg DMB; 95% CI = 3.5 mg/kg DMB, 13.1 mg/kg DMB). Thiamin content was 49.8% higher (*P* < 0.05) when formulas contained LBV (40.3 mg/kg DMB; 95% CI = 31.0 mg/kg DMB, 50.9 mg/kg DMB) than formulas that contained BY (26.9 mg/kg DMB; 95% CI = 19.4 mg/kg DMB, 35.7 mg/kg DMB). Formulas that contained EA (19.3 mg/kg DMB; 95% CI = 13.0 mg/kg DMB, 26.8 mg/kg DMB) or NY (19.3 mg/kg DMB; 95% CI = 13.1 mg/kg DMB, 26.8 mg/kg DMB) were not different (*P* > 0.05) from each other. However, formulas that included either NY or EA contained 52.1% and 28.2% less (*P* < 0.05) thiamin than when LBV or BY, respectively, were included in the formula.

Thiamin decline during 6 months of storage was not linear (*P* > 0.05; Figure 3). Instead, the quadratic and cubic contrasts (*P* < 0.05) were both significant. However, thiamin content was not different (*P* > 0.05) at the beginning (month 0 average = 23.4 mg/kg DMB; 95% CI = 15.6 mg/kg DMB, 32.7 mg/kg DMB), middle (month 3 average = 23.3 mg/kg DMB; 95% CI = 15.5 mg/kg DMB, 32.6 mg/kg DMB), or the end of storage (month 6 average = 24.6 mg/kg DMB; 95% CI = 16.6 mg/kg DMB, 34.1 mg/kg DMB). However, the greatest (*P* < 0.05) thiamin content was observed in month 1 (average = 30.1 mg/kg DMB; 95% CI = 21.1 mg/kg DMB, 40.6 mg/kg DMB) and a 12.4% increase (*P* < 0.05) was observed in months 4 (average = 26.0 mg/kg DMB; 95% CI = 17.7 mg/kg DMB, 35.8 mg/kg DMB)) and 5 (average = 26.4 mg/kg DMB; 95% CI = 18.1 mg/kg DMB, 36.3 mg/kg DMB) compared to month 3.

**Figure 4:**
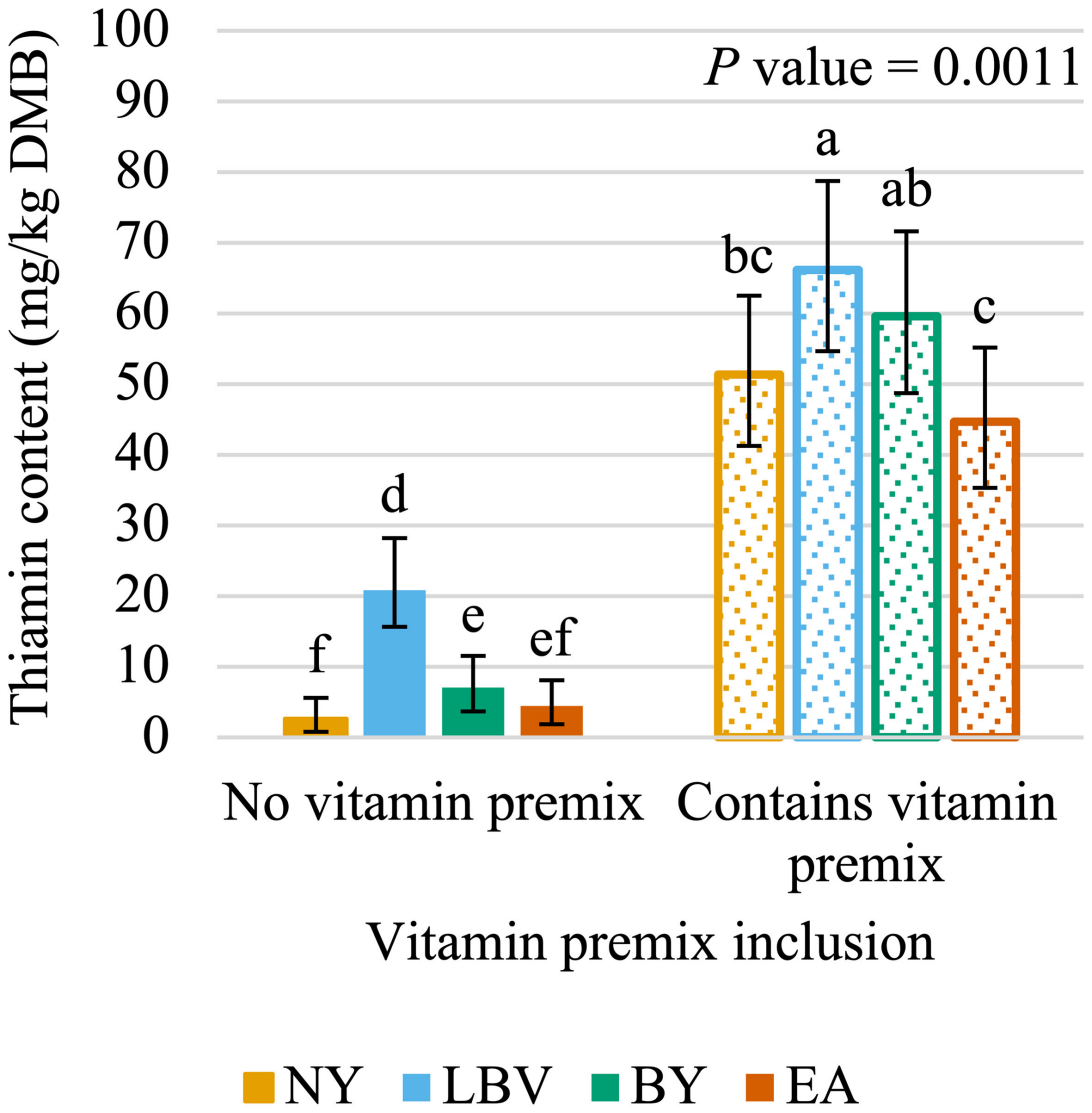
Interaction of vitamin premix inclusion and yeast inclusion^1^ on dry matter basis (DMB) thiamin content (average with 95% confidence interval) of canned cat food stored in a commercial warehouse for 6 months. ^abcdef^ Means without a common superscript are different (*P* < 0.05). ^1^NY, no yeast; LBV, Lalmin B-complex vitamins; BY, spray dried brewer's yeast #1064B; EA, BGYADVANTAGE.

The interaction of vitamin premix inclusion and yeast inclusion was significant (*P* < 0.05; Figure 4). The diet containing LBV and the vitamin premix (66.1 mg/kg DMB; 95% CI = 54.6 mg/kg DMB, 78.7 mg/kg DMB) and the diet containing BY and the vitamin premix (59.6 mg/kg DMB; 95% CI = 48.7 mg/kg DMB, 71.6 mg/kg DMB) contained 47.2% and 33.4%, respectively, more (*P* < 0.05) thiamin than the formula with EA and the vitamin premix (44.9 mg/kg DMB; 95% CI = 34.3 mg/kg DMB, 55.1 mg/kg DMB). The DMB thiamin content of the diet containing NY and the vitamin premix (51.3 mg/kg DMB, 95% CI = 41.3 mg/kg DMB, 62.5 mg/kg DMB) was not different (*P* > 0.05) than of diets containing the vitamin premix and either BY or EA. However, the NY and vitamin premix diet did contain 22.4% less (*P* < 0.05) thiamin than the LBV and vitamin premix diet. All diets including the vitamin premix contained more (*P* < 0.05) thiamin than all diets that did not include the vitamin premix, regardless of yeast inclusion. The diet with LBV and no vitamin premix (20.9 mg/kg DMB; 95% CI = 14.6 mg/kg DMB, 28.2 mg/kg DMB) contained more (*P* < 0.05) thiamin than the diets containing no vitamin premix and either BY (7.0 mg/kg DMB, 95% CI = 3.7 mg/kg DMB, 11.5 mg/kg DMB), EA (4.4 mg/kg DMB; 95% CI = 1.9 mg/kg DMB, 8.1 mg/kg DMB), or NY (2.7 mg/kg DMB; 95% CI = 0.8 mg/kg DMB, 5.6 mg/kg DMB). Including BY without the vitamin premix resulted in 2.66 times as much (*P* < 0.05) thiamin as NY without the vitamin premix. The diet containing EA without the vitamin premix had similar (*P* > 0.05) levels of thiamin compared to diets without the vitamin premix and containing either NY or BY.

**Figure 5:**
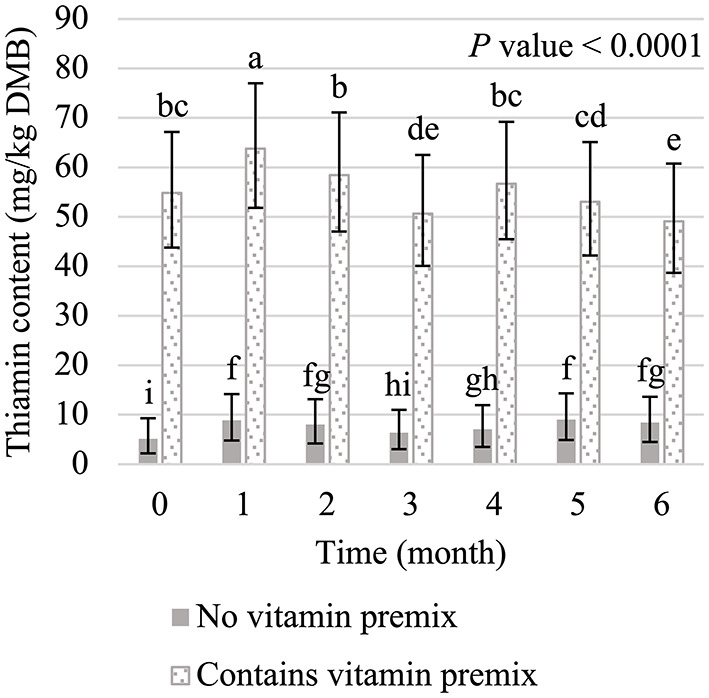
Interaction of vitamin premix inclusion and time on dry matter basis (DMB) thiamin content (average with 95% confidence interval) of canned cat food stored in a commercial warehouse for 6 months. ^a−i^Means without a common superscript are different (*P* < 0.05).

The interaction of vitamin premix and time was significant (*P* < 0.05; Figure 5). Thiamin content increased (*P* < 0.05) during storage when vitamin premix was not included in the diet; a 64.7% increase in thiamin content was observed between month 0 and month 6. However, thiamin levels for all diets without vitamin premix were low and averages ranged from 5.1 mg/kg DMB to 9.0 mg/kg DMB. Thiamin content also fluctuated slightly when vitamin premix was included in the diet. The highest content was observed in month 1 (average = 63.8 mg/kg DMB; 95% CI = 51.8 mg/kg DMB, 77.0 mg/kg DMB) and the lowest content in month 6 (average = 49.1 mg/kg DMB; 95% CI = 38.7 mg/kg DMB, 60.8 mg/kg DMB). Overall, thiamin content decreased (*P* < 0.05) by 10.6% when month 0 (average = 54.9 mg/kg DMB; 95% CI = 43.8 mg/kg DMB, 67.2 mg/kg DMB) and month 6 were compared.

**Figure 6:**
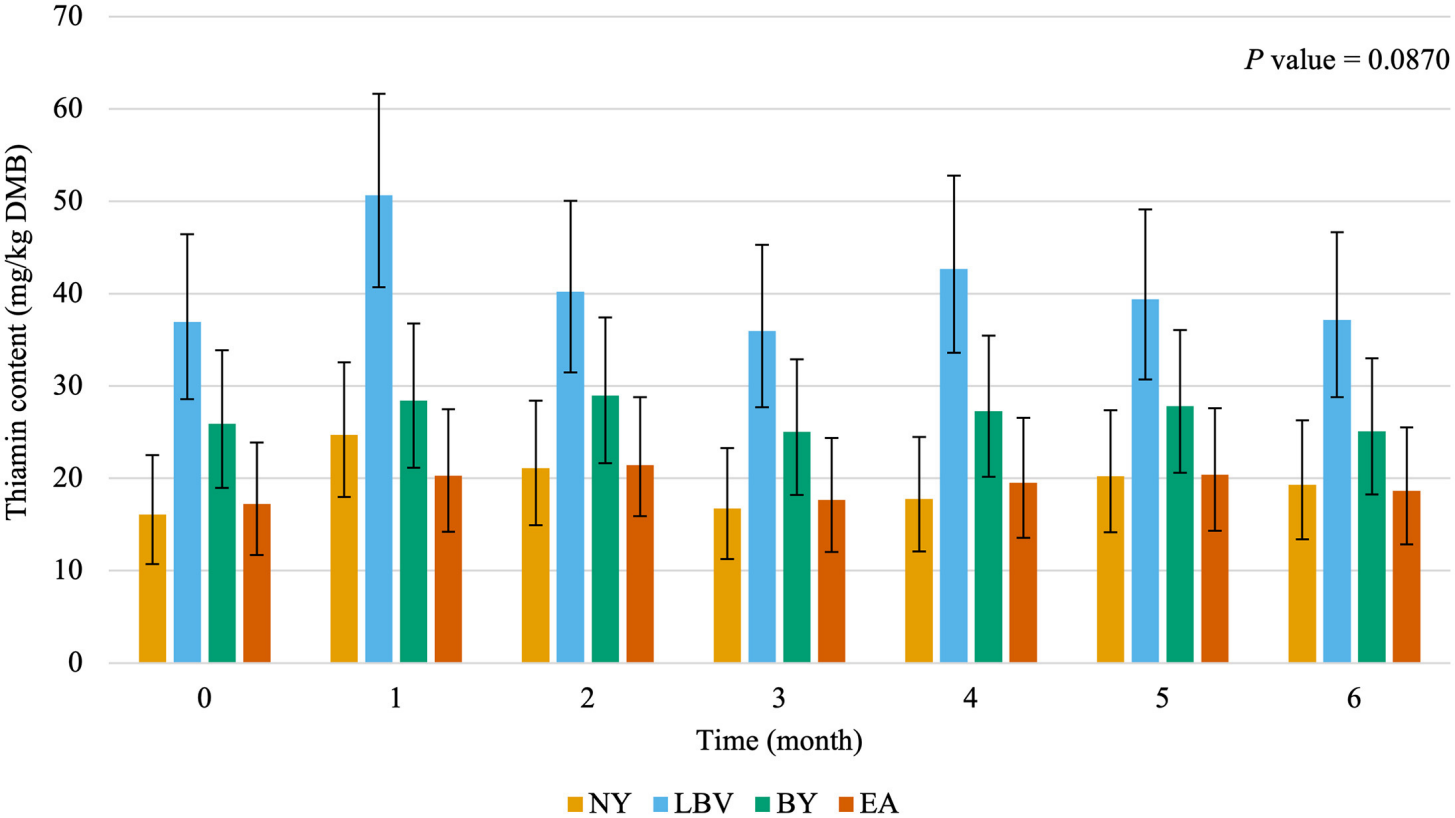
Interaction of yeast inclusion^1^ and time on dry matter basis (DMB) thiamin content (average with 95% confidence interval) of canned cat food stored in a commercial warehouse for 6 months. ^1^NY, no yeast; LBV, Lalmin B-complex Vitamins; BY, spray dried brewer's yeast #1064B; EA, BGYADVANTAGE.

The interaction of yeast and time was not significant (*P* > 0.05; Figure 6). Average thiamin content across yeast inclusions during the 6 months of storage was 26.5 mg/kg DMB.

The interaction of vitamin premix, yeast, and time was significant (*P* < 0.05; Supplementary Figure 1). The diets containing LBV or BY without vitamin premix and diets containing BY or EA with vitamin premix did not (*P* > 0.05) exhibit thiamin degradation during 6 months of storage. The highest (*P* < 0.05) level of thiamin in the diet with no vitamin premix and no yeast was observed in months 1, 5, and 6 (average = 4.6 mg/kg DMB) and the lowest (*P* < 0.05) level in month 0 (average = 0.5 mg/kg DMB; 95% CI = 0.0 mg/kg DMB, 2.2 mg/kg DMB). This represented a >8-fold increase in thiamin content on a very small starting base. Thiamin content of the diet containing EA without vitamin premix was lower (*P* < 0.05) at month 0 (average = 5.8 mg/kg DMB; 95% CI = 2.7 mg/kg DMB, 10.1 mg/kg DMB) than at months 1, 2, 5, and 6 (average = 7.8 mg/kg DMB), representing a 34.5% increase. The diet containing vitamin premix with no yeast had less (*P* < 0.05) thiamin after 6 months of storage with a 17.2% decrease. Thiamin content in the diet containing vitamin premix and LBV was higher (*P* < 0.05) in month 1 (average = 89.6 mg/kg DMB; 95% CI = 75.7 mg/kg DMB, 104.7 mg/kg DMB) than in any other month of storage (average = 62.7 mg/kg DMB). Replicates of this diet had a wider range of thiamin contents (54.2 mg/kg DMB to 126.9 mg/kg DMB) compared to replicates at other sample collection times.

## Discussion

The aim of this work was to identify yeast ingredients as potential thiamin sources and to evaluate the stability of their intrinsic thiamin during storage of 6 months in a commercial warehouse. Preliminary studies identified that retort processing conditions (10), package size and material (9), protein source (11), and the presence of sulfites (11) influence thiamin content in canned cat food. However, none of those experiments identified a solution to minimize thiamin degradation that could be applied to all thermally processed cat foods. If a yeast ingredient had similar or better shelf life compared to thiamin mononitrate, then it could be a suitable alternative thiamin source.

### Macronutrient and mineral contents

Commercial cat foods contain moisture ranging from 78 to 82% maximum (15, 16). The diets in the present experiment fall just outside the upper end of that range. Data from a pilot study suggested that pre-retort batter moisture content could influence the concentration of thiamin after retort processing (10). However, all 8 diets contained similar levels of moisture, which likely did not influence thiamin content during storage differently. Future experiments could achieve lower moisture contents by decreasing water and/or steam addition in a production setting.

All 8 diets met the AAFCO crude protein (26% DMB) and crude fat (9% DMB) requirements for adult cats (2). In general, crude protein content was higher for diets containing BY or EA vs. NY. This is likely due to the nutritional composition of the yeast ingredients and the brewer's rice used as a space-holding ingredient. The brewer's rice contained 9.2% DMB crude protein whereas the three yeast ingredients contained an average of 53.5% DMB crude protein (12). Diets containing LBV were intermediate in terms of crude protein content; this is likely due to the ingredient's lower inclusion level compared to BY and EA. This influenced the differences observed in NFE content, a calculation which typically accounts for starches, sugars, and some non-starch polysaccharides (17). Diets containing BY and EA contained less NFE compared to diets containing NY with diets containing LBV in the middle. Interestingly, diets containing EA contained slightly less NFE than diets containing BY. This was likely influenced by the slightly higher crude fat and crude fiber contents of the two ingredients that were not as detectable in the complete diets.

Similarly, all 8 diets met the AAFCO minimum requirements for the minerals analyzed in the experiment. While this is not unexpected, it is meaningful to note that inclusion of yeast ingredients as thiamine sources did not affect the formulas' abilities to meet minimum levels. Differences in the minerals contents of finished diets could be attributed to the mineral contents of the yeast ingredients themselves, as ground brewer's rice made relatively little impact (12).

Limited data are available on typical macronutrient and mineral contents of commercial canned cat foods. Reports of the macronutrient content of three commercial products suggested the typical diet may range from 41.3 TO 51.5% DMB crude protein and 33.9–40.7% DMB crude fat by acid hydrolysis (18–20). Two studies reported ash contents between 7.2 and 11.05% DMB (19, 20). Only one study reported crude fiber and NFE contents (1.2–1.5% DMB and 2.0–5.2% DMB, respectively) for a diet across a longitudinal feeding study (19). Regardless, the diets in the present experiment contained similar nutrient levels, which suggested that they were fair representations of commercially available diets.

### Thiamin content during storage

It was expected that the main effects of vitamin premix and yeast and their interaction would be significant due to the experimental design. However, it was not expected that inclusion of EA would result in similar thiamin levels compared to inclusion of NY when the effects of vitamin premix and time were not considered. Thiamin degradation during thermal processing of these canned cat foods was greater for EA than NY when vitamin premix inclusion was not considered (12). This likely caused their thiamin contents to be similar even though the expectation was that inclusion of EA would result in similar thiamin content compared to the inclusion of BY instead.

This is the first experiment to assess the storage stability of thiamin in canned pet food. The storage length of 6 months represented the first 25% of a canned cat food's shelf life. During this time, canned cat foods may be transported from the manufacturing facility to a warehouse or distribution center before they are received by a retailer (21). While it is worthwhile to conduct an extended storage study to represent the entire shelf life of a canned cat food, this fell outside the scope of the present experiment.

The cubic relationship for the main effect of time was somewhat expected given the sensitivity of the high performance liquid chromatography methodology employed for thiamin analysis. For example, the relative standard deviation of thiamin analysis of canned cat food within select laboratories was 4.302%, whereas the value for riboflavin analysis in canned cat food was 1.687% (22). Measures can be taken to minimize variability in the assay, such as the use of amber glassware to prevent light degradation of the thiamin (23). Additionally, all samples in this experiment were analyzed by the same commercial laboratory to eliminate variation between laboratories as a contributing factor. Composite samples of three cans per treatment-timepoint combination were analyzed instead of single cans to minimize sampling variation. Therefore, it can be assumed that the variability observed is representative of the sensitivity of the assay and minimally confounded by other influences. This variability likely influenced the statistically observed cubic relationship between storage time and thiamin content. Other statistical observations likely influenced include the increase in thiamin content of the diets containing no vitamin premix and no yeast or EA compared to month 0, which would influence the statistical conclusions made regarding the interaction between storage time and the absence of vitamin premix. An extended storage study as suggested early in the manuscript may allow for a better picture of this main effect between thiamin content and storage time.

Storage of extruded pet food found that 80% of thiamin was retained after 6 months of storage when thiamin mononitrate was the sole thiamin source (13). This was similar to the 82.2% thiamin retention observed in the present experiment in the diet containing a standard vitamin premix with thiamin mononitrate and no yeast ingredient. This implied that supplemental thiamin degradation during storage is similar regardless of food format and could be confirmed by a future experiment evaluating the degradation of thiamin mononitrate during storage in food processed by extrusion, canning, baking, and other processes. However, other researchers did not observe differences in thiamin content of commercial extruded diets for cats after 6 months of storage (24). Thiamin mononitrate and thiamin hydrochloride are the most common supplemental thiamin sources included in pet food formulations (23). Therefore, it can be assumed that those diets utilized one of those sources but cannot be confirmed as the authors did not disclose the ingredients in each diet. This could have significantly influenced thiamin retention and further stresses the importance of diet formulation as a factor in thiamin retention. Storage of canned rainbow trout and canned pollock identified greater retention in the rainbow trout than the pollock after 6 and 12 months of storage at 22°C (25). Thiamin intrinsic to meat may have less thermal stability compared to other ingredients. Thermally processed beef brisket retained roughly 33% of its post-processing thiamin content after 6 months of storage at 20°C, while thiamin retention for brown rice and split pea soup stored in the same conditions ranged from 85 to 95% (26). The other ingredients in the canned diet can influence thiamin retention even when meat is the primary ingredient. Care should be taken to minimize the oxidation level of fats and/or include antioxidants in the formula. For example, use of fresh fats and the inclusion of casein hydrolysate or rosemary extract improved thiamin retention in sterilized chicken meat during storage (27). Antioxidants were not included in canned cat food in this experiment to mimic a worst-case scenario. Additional fat inclusion was not necessary as the mechanically deboned low ash chicken (50.2% DMB crude fat), frozen pork liver (24.5% DMB crude fat), and ground chicken (46.2% DMB crude fat) (12) provided sufficient levels of crude fat to meet nutritional requirements of adult cats. Nevertheless, the addition of pork lard to chicken meat resulted in a longer half-life of thiamin during storage compared to the addition of soy or sunflower oil (27). It is possible that more thiamin could have been degraded if an unsaturated fat source was included in the diets, but this fell outside the scope of the present experiment.

The intent of this experiment was not to identify the mechanism behind observed differences in thiamin content of the canned cat foods. However, it is likely that the different thiamin forms present in thiamin mononitrate and the evaluated yeast ingredients greatly affected their storage stability. Previous researchers have identified thiamin binding proteins, which provided a protective effect on the thiamin during early stages of the yeast cells (28). The presence of thiamin binding proteins in the evaluated yeast ingredients could explain why diets containing them relatively maintained their thiamin content during storage. Confirmation of the presence of thiamin binding proteins in yeast ingredients fell outside the scope of this experiment, but could be a useful analysis when screening yeast ingredients as thiamin sources.

## Conclusions

The present experiment identified 3 yeast ingredients that stabilized thiamin content in canned cat food during 6 months of storage in a commercial warehouse. In comparison, thiamin content of a diet containing a thiamin mononitrate from a vitamin premix decreased by 17.8% when stored in the same conditions. Further research is needed to discover how thiamin content is affected by prolonged storage to the end of shelf-life as well as how other formulation factors could affect thiamin stability. However, the data herein were promising and suggested that canned cat food formulas should consider including a yeast ingredient to promote better thiamin stability during storage.

## Data availability statement

The raw data supporting the conclusions of this article will be made available by the authors, without undue reservation.

## Author contributions

CA and AD designed the experiment. AD conducted the research, performed the statistical analyses, and wrote the manuscript. All authors revised and provided intellectual input on this manuscript. All authors contributed to the article and approved the submitted version.
