# Supplementary material for: Impacts of vitamin premix and/or yeast ingredient inclusion in a canned cat food on thiamin retention during 6 months of storage
Source: Front Vet Sci. 2022 Dec 8;9:1090695. doi: 10.3389/fvets.2022.1090695 (PMC9771992; doi:10.3389/fvets.2022.1090695)
Supplement: Supplementary file 1 [file Table_1.DOCX]

Supplementary Material

# Supplementary Table 1: Macronutrient and mineral composition (mean ± standard deviation) of canned cat foods containing different levels of a vitamin premix and/or a yeast ingredient1 averaged over 6 months of storage in a commercial warehouse.

|  | No vitamin premix | | | | | Contains vitamin premix | | | |
| --- | --- | --- | --- | --- | --- | --- | --- | --- | --- |
| Nutrient | NY | LBV | BY | EA | NY | | LBV | BY | EA |
| Moisture, % | 82.8 ± 0.86 | 82.7 ± 0.99 | 83.1 ± 0.80 | 82.1 ± 0.99 | 82.6 ± 0.91 | | 82.4 ± 1.01 | 82.9 ± 0.78 | 82.1 ± 0.96 |
|  | ------------------------------------------------------------ Dry matter basis ------------------------------------------------------------ | | | | | | | | |
| Crude protein, % | 38.2 ± 1.80 | 40.2 ± 2.37 | 50.4 ± 1.90 | 49.9 ± 3.11 | 38.7 ± 3.93 | | 40.0 ± 3.14 | 51.0 ± 3.48 | 49.5 ± 2.83 |
| Crude fat, % | 30.1 ± 4.68 | 30.2 ± 4.90 | 26.9 ± 3.37 | 30.4 ± 4.00 | 30.9 ± 5.65 | | 30.2 ± 4.90 | 29.1 ± 3.18 | 30.5 ± 4.27 |
| Crude fiber, % | 0.74 ± 0.316 | 0.67 ± 0.277 | 1.24 ± 0.368 | 1.70 ± 0.230 | 0.93 ± 0.296 | | 0.86 ± 0.256 | 1.34 ± 0.451 | 1.83 ± 0.271 |
| Ash, % | 5.51 ± 1.343 | 5.91 ± 1.220 | 6.43 ± 1.447 | 7.31 ± 1.383 | 5.99 ± 1.407 | | 6.15 ± 1.417 | 6.42 ± 1.141 | 7.70 ± 1.293 |
| NFE^2^, % | 25.5 ± 2.62 | 23.0 ± 3.05 | 12.4 ± 2.15 | 10.7 ± 2.65 | 23.5 ± 4.47 | | 22.9 ± 3.44 | 12.2 ± 3.96 | 10.5 ± 3.50 |
| Calcium, % | 1.25 ± 0.259 | 1.27 ± 0.232 | 0.96 ± 0.297 | 1.42 ± 0.266 | 1.33 ± 0.293 | | 1.34 ± 0.284 | 1.04 ± 0.313 | 1.46 ± 0.230 |
| Phosphorus, % | 0.982 ± 0.1494 | 1.020 ± 0.1346 | 1.150 ± 0.1564 | 1.153 ± 0.1547 | 1.017 ± 0.1569 | | 1.029 ± 0.1539 | 1.145 ± 0.1602 | 1.151 ± 0.1407 |
| Potassium, % | 0.797 ± 0.1679 | 0.880 ± 0.1772 | 1.184 ± 0.1560 | 1.352 ± 0.1947 | 0.810 ± 0.2053 | | 0.859 ± 0.1759 | 1.155 ± 0.1682 | 1.342 ± 0.1818 |
| Sodium, % | 0.318 ± 0.0423 | 0.321 ± 0.0460 | 0.324 ± 0.0367 | 0.312 ± 0.0453 | 0.316 ± 0.0458 | | 0.314 ± 0.0463 | 0.324 ± 0.0436 | 0.312 ± 0.0424 |
| Magnesium, % | 0.072 ± 0.0080 | 0.075 ± 0.0067 | 0.101 ± 0.0081 | 0.142 ± 0.0108 | 0.074 ± 0.0122 | | 0.074 ± 0.0074 | 0.098 ± 0.0091 | 0.141 ± 0.0120 |
| Sulfur, % | 0.45 ± 0.052 | 0.46 ± 0.050 | 0.56 ± 0.049 | 0.52 ± 0.054 | 0.45 ± 0.059 | | 0.45 ± 0.051 | 0.55 ± 0.046 | 0.52 ± 0.053 |
| Iron, mg/kg | 265 ± 76.9 | 276 ± 87.0 | 241 ± 76.3 | 293 ± 81.6 | 272 ± 82.9 | | 268 ± 79.7 | 245 ± 78.6 | 289 ± 74.5 |
| Copper, mg/kg | 32.95 ± 8.711 | 33.72 ± 9.536 | 39.23 ± 8.897 | 36.50 ± 9.447 | 33.15 ± 8.951 | | 32.59 ± 8.963 | 38.61 ± 9.619 | 36.28 ± 8.838 |
| Manganese, mg/kg | 19.27 ± 6.048 | 19.73 ± 6.121 | 12.69 ± 7.735 | 25.88 ± 6.770 | 19.86 ± 6.338 | | 19.71 ± 6.186 | 14.41 ± 7.001 | 26.38 ± 6.238 |
| Zinc, mg/kg | 177 ± 52.3 | 183 ± 52.3 | 172 ± 59.7 | 187 ± 55.2 | 181 ± 54.8 | | 182 ± 53.2 | 174 ± 59.5 | 184 ± 50.8 |
| ^1^ NY = no yeast; LBV = Lalmin B-Complex Vitamins; BY = spray-dried brewer’s yeast #1064B; EA = BGYADVANTAGE.  ^2^ NFE = nitrogen free extract, calculated (Dry matter basis contents of crude protein, crude fat, crude fiber, and ash subtracted from 100). | | | | | | | | | |


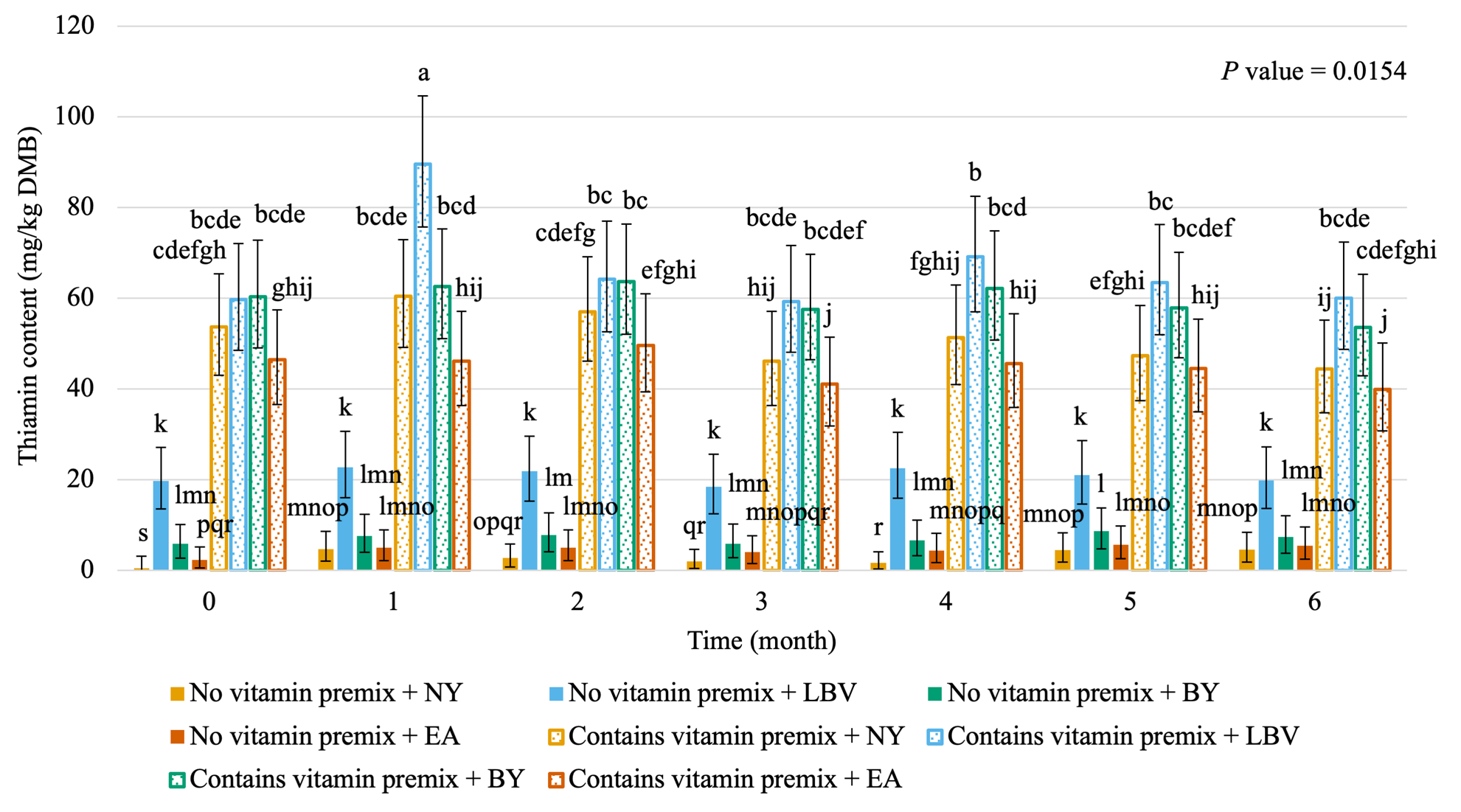


**Supplementary Figure 1: Interaction of vitamin premix inclusion, yeast inclusion^1^, and time on dry matter basis (DMB) thiamin content (average with 95% confidence interval) of canned cat stored in a commercial warehouse for 6 months.** ^abcdefghijklmnopqrs^ Means without a common superscript are different (*P* < 0.05). ^1^ NY = no yeast; LBV = Lalmin B-complex Vitamins; BY = spray dried brewer’s yeast #1064B; EA = BGYADVANTAGE.
